# Size and Reproductive Traits Rather than Leaf Economic Traits Explain Plant-Community Composition in Species-Rich Annual Vegetation along a Gradient of Land Use Intensity

**DOI:** 10.3389/fpls.2017.00891

**Published:** 2017-05-29

**Authors:** Inga Dirks, Rita Dumbur, Patrick Lienin, Michael Kleyer, José M. Grünzweig

**Affiliations:** ^1^Robert H. Smith Institute of Plant Sciences and Genetics in Agriculture, Robert H. Smith Faculty of Agriculture, Food and Environment, The Hebrew University of JerusalemRehovot, Israel; ^2^Landscape Ecology Group, University of OldenburgOldenburg, Germany

**Keywords:** biodiversity, disturbance, land use intensity, Mediterranean ecosystems, plant functional groups, plant functional traits, soil resources, trait-environment relationships

## Abstract

Agricultural land use imposes a major disturbance on ecosystems worldwide, thus greatly modifying the taxonomic and functional composition of plant communities. However, mechanisms of community assembly, as assessed by plant functional traits, are not well known for dryland ecosystems under agricultural disturbance. Here we investigated trait responses to disturbance intensity and availability of resources to identify the main drivers of changes in composition of semiarid communities under diverging land use intensities. The eastern Mediterranean study region is characterized by an extended rainless season and by very diverse, mostly annual communities. At 24 truly replicated sites, we recorded the frequency of 241 species and the functional traits of the 53 most common species, together with soil resources and disturbance intensity across a land use gradient ranging from ungrazed shrubland to intensively managed cropland (six land use types). Multivariate RLQ analysis (linking functional traits, sites and environmental factors in a three-way ordination) and fourth corner analysis (revealing significant relations between traits and environmental factors) were used in a complementary way to get insights into trait-environment relations. Results revealed that traits related to plant size (reflecting light absorption and competitive ability) increased with resource availability, such as soil phosphorus and water holding capacity. Leaf economic traits, such as specific leaf area (SLA), leaf nitrogen content (LNC), and leaf dry matter content showed low variation across the disturbance gradient and were not related to environmental variables. In these herbaceous annual communities where plants grow and persist for just 3–5 months, SLA and LNC were unrelated, which together with relatively high SLA values might point to strategies of drought escape and grazing avoidance. Seed mass was high both at higher and lower resource availability, whereas seed number increased with the degree of disturbance. The strong response of size and reproduction traits, and the missing response of leaf economic traits reveal light interception and resource competition rather than resource acquisition and litter decomposition as drivers of plant community composition. Deviations from trait relationships observed in commonly studied temperate ecosystems confirm that climatic conditions play a fundamental role by filtering species with particular life forms and ecological strategies.

## Introduction

Large parts of the terrestrial surface are exposed to human-caused land use change that brings major disturbances to natural ecosystems. High frequency and intensity of agricultural disturbances, such as plowing, weed control, input of nutrients through fertilization, combustion of biomass, and cattle grazing severely modify the composition of plant communities worldwide. Among components of global change, land use change was identified as the largest driver of decrease in biodiversity and in modification of plant communities (Sala et al., 2000).

The Mediterranean-type regions of the globe are hotspots of biodiversity for their large number of plant species and their high degree of endemism (Shmida, 1984; Medail and Quezel, 1997). Under the conditions of the characteristic summer drought and following 1000s of years of human land use and disturbance by grazing, fire and additional activities, a specific vegetation evolved in Mediterranean regions that is dominated by a very diverse annual community, often associated with a few woody species (Naveh and Whittaker, 1979; Cowling et al., 1996). Both diverse land use activities and summer droughts shorten the timeframe for growth and reproduction, effectively advantaging the annual strategy in these regions. Consequently, Mediterranean ecosystems are mainly composed of winter annuals that germinate after the first autumn rains, flower and set seed during spring, die at the onset of the summer season, and pass the hot and dry summer season as seeds in the soil (Fernández Ales et al., 1993).

Disturbance can be defined as the partial or total destruction of biomass, which might occur more than once during a growing season (Grime, 2001). Thus, land use affects plants to various degrees, depending on its intensity ranging from low disturbance by light grazing to complete removal of vegetation (above- and belowground) by intensive cropping activities, such as plowing (Harper, 1977). Responses of communities to disturbance are associated with variation in life history, plant size, architecture, leaf functioning, resprouting capacity, and seed traits, which are induced by a modification in species composition (Bond and Midgley, 2001; McIntyre and Lavorel, 2001; McIntyre, 2008).

The response of plant communities to disturbance can be assessed by an analysis of plant functional traits, which comprise information about plant size, leaf morphology, leaf chemical composition, and reproduction. Functional traits pertaining to size (‘size traits’) describe space utilization and pre-emption, suggesting light interception and competitive ability as drivers of community composition. ‘Leaf economic traits’ characterize nutrient and water acquisition, growth rate and turnover of resources (Wright et al., 2004), which can also be linked to litter decomposability (Lavorel et al., 2007). High leaf nutrient concentrations and short-lived leaves are positively associated with resource acquisition and high site fertility across various biomes (Reich et al., 1992; Grime, 2001; Wright et al., 2004; Poorter and Garnier, 2007, Osnas et al., 2013), including Mediterranean old-field communities (Violle et al., 2007). Opposite trait values characterize species from nutrient-poor habitats (Elser et al., 2010). Finally, ‘reproductive traits’ characterize the reproductive strategy, with larger seed mass (SM) favoring seedling germination, growth and survival, and possibly acting as a buffer against poor environmental conditions (Pakeman et al., 2008). Large seed output increases the number of colonization opportunities, which is of importance in disturbed landscapes (Leishman, 2001). An inverse relationship between SM and seed number (SN) was often observed (e.g., Jakobsson and Eriksson, 2000; Kühner and Kleyer, 2008). Altogether, these traits represent relevant axes of specialization of plants in their environment.

Functional traits can be helpful to understand fundamental ecosystem functions, such as light interception, absorption of nutrients and water, nutrient retention, carbon fluxes, competitive ability and dispersal strategies (Lavorel et al., 2007). These ecosystem functions, in turn, hold information about drivers of plant community composition, such as disturbance and soil resources. However, those drivers are still unclear regarding annual herbaceous vegetation in the drought affected semiarid Mediterranean vegetation. To understand which biological functions, such as growth, leaf function, and regeneration are most strongly affected by land use change, trait expression of co-occurring species can be linked to environmental conditions on gradients of land use intensity (Lienin and Kleyer, 2011). Importantly, a certain land use type might have different consequences for functional traits under diverging environmental conditions. Grazing effects, for example, depend on the variability of precipitation and season (Milchunas et al., 1988). Under moist conditions, grazing was related to high specific leaf area and leaf nitrogen content as grazing-tolerance traits (Adler et al., 2004; Cingolani et al., 2005), whereas defense traits, such as spines on leaves imply small leaf size and high dry matter content at grazed sites in dry climates (Wardle et al., 1998; Díaz and Cabido, 2001). Thus, leaf economic traits can possibly deviate from the leaf economics spectrum (Wright et al., 2004) in certain environments.

By using plant functional traits we aimed at detecting strategies of annual plants to cope with disturbance caused by land use in combination with soil resource availability. The determination of the most responsive functional traits should improve the understanding of the critical drivers of community composition in the semiarid eastern Mediterranean ecosystems under land use intensification against the background of extreme drought. We pursued the following objectives in this study: (1) to detect the variation in functional traits and resources across an extended land use gradient; (2) to relate traits to the main environmental factors that change in the landscape (trait-environment relationships); (3) to reveal strategies of annual plants to cope with disturbance in combination with resource availability by defining plant functional groups. Plant functional traits representing plant size, leaf morphology, leaf chemical composition and reproduction of the most common annual species were systematically determined at replicated field sites chosen to maximize variability in disturbance intensity and soil resources across a species-rich Mediterranean landscape. Increasing land use intensity in annual herbaceous vegetation presumably leads to increasing pressure to complete the plants’ life cycle, which would be expressed by (1) higher investment in traits associated with fast growth and biomass acquisition, and (2) higher investment in leaf economic traits. A shift in functional traits as affected by agricultural land use will further imply shifts in dispersal strategies, with high SNs increasing the opportunity for colonization (Leishman, 2001) and larger SM favoring seedling germination, growth and survival (Pakeman et al., 2008; Metz et al., 2010). Therefore, we hypothesized (3) higher investment in dispersal units (SM and SN) with increasing land use intensity.

## Materials and Methods

### Field Sites and Study Plots

In total, 24 field sites were surveyed in the northern part of the Judean Foothills (31°36′-31°39′ N, 34°50′-34°56′ E, ∼35 km from the Mediterranean coast at 225–345 m above sea level), a dry sub-humid Mediterranean region in central Israel (Supplementary Figure A1). The climate is dominated by cool and moist winters, and hot and dry summers, with a rainless period between May and September or October. Mean annual precipitation is 480 mm, mean annual temperature is 20.2°C. The geology of the Judean Foothills is characterized by chalk and hard limestone crusts covered with Rendzina and related soil types. The natural vegetation is composed of (i) shrubland, with species-rich, mostly annual vegetation and scattered shrubs and dwarf shrubs; and (ii) grassland composed of species-rich, mostly annual vegetation (Shachak et al., 2008; Dirks et al., 2010).

The field sites were selected to represent the six main land use types of the Judean Foothills, along a general gradient of increasing disturbance intensity from ungrazed and grazed shrublands, to grasslands, olive groves and intensively cultivated legume and cereal cropland (**Table 1**). Plant communities in olive groves and cropland comprise ruderal species and remnants of the more complex herbaceous communities. Shrubland in this study was either ungrazed (but browsed by wild animals) or subjected to moderate grazing by sheep or cattle. Grassland was moderately or heavily grazed by sheep or cattle. Olive groves and cropland were exposed to mechanical cultivation and/or chemical weed control.

**Table 1:** Environmental characterization of each land use type as used in the multivariate analyses.

Land use type	No. of sites	Disturbance index	Species richness per m^2^	Soil carbon-to-nitrogen ratio	Soil organic carbon stock (kg m^-2^)	Soil phosphorus availability (μg P ml^-1^)	Soil water holding capacity (m^3^ H_2_O m^-3^ soil)
Shrubland, ungrazed	4	0.05 (0.01–0.10)	65 (57–79)	10.9 (10.3–11.8)	3.42 (2.58–4.88)	0.18 (0.14–0.24)	0.37 (0.35–0.39)
Shrubland, grazed	3	0.28 (0.18–0.40)	60 (35–86)	11.6 (9.36–13.3)	2.21 (1.53–2.86)	0.30 (0.10–0.65)	0.35 (0.31–0.40)
Grassland	5	0.37 (0.20–0.50)	52 (36–65)	12.3 (10.4–18.2)	2.64 (2.03–3.51)	0.74 (0.26–2.46)	0.37 (0.28–0.42)
Olive grove	2^∗^	1.25 (1.10–1.50)	31 (16–40)	18.2 (11.1–24.7)	2.03 (1.74–2.58)	0.53 (0.10–0.71)	0.40 (0.38–0.42)
Cropland, legume	5	1.92 (1.60–2.10)	7 (2–14)	14.8 (11.9–21.8)	0.86 (0.44–1.94)	0.25 (0.06–0.51)	0.43 (0.39–0.45)
Cropland, cereal	5	2.00 (1.50–2.50)	5 (3–9)	20.1 (13.2–38.1)	1.25 (1.13–1.35)	0.79 (0.33–1.60)	0.40 (0.36–0.46)

*^∗^Means include data from two study plots per site, four in total for olive groves (see “Materials and Methods”). All other land use types had one study plot per site.*

*Shown are means, with range of values in parentheses.*

Each land use type was replicated by 2–5 field sites, with one study plot of 10 m × 10 m employed at all sites, except of olive groves where two plots were installed (see below). In shrubland and olive groves, only herbaceous patches were considered here, and shrubs and trees with very different traits and life history were excluded from the analysis. Management of olive groves within rows of olive trees (mown twice a year) differed from management of the area between rows of trees (plowed and additionally mown). Because of the difference in disturbance intensity two study plots were installed in each olive grove, one within and one between rows of trees. These plots in the two olive groves were treated separately in the analyses.

### Disturbance Index

Disturbance was characterized by a measure of biomass removal intensity (see Kleyer, 1999; White and Jentsch, 2001), as follows:

(1) $$\text{Dist}=\sum F_{i}*I_{i}$$

where *F* = frequency of disturbance event *i* per year; *I* = magnitude of disturbance event *i*, expressed as relative biomass removal. For instance, a field may be plowed twice a year (*F* = 2) which destroys all plants, including roots (*I* = 1). Disturbance frequency and magnitude in olive groves and croplands was recorded by interviewing the respective farmers. For grazing, disturbance magnitude could not be derived from cultivation routines because cattle and sheep ranged more or less freely over the area during grazing. Therefore, disturbance induced by grazing was quantified as follows:

(2) $$I=({\text{B}}_{\text{no}\_\text{graz}}{\text{-B}}_{\text{graz}})/{\text{B}}_{\text{no}\_\text{graz}}$$

where B_no_graz_ = total biomass in an ungrazed patch and B_graz_ = total biomass in a grazed patch. In all cases, grazing was conducted once during the grazing season (*F* = 1). Grazing did not disturb belowground buds, roots or rhizomes, but could in an extreme case consume all aboveground biomass (*I* = 0.5). We assumed a belowground:aboveground ratio (‘root:shoot ratio’) of 1 in the annual vegetation of the Judean Foothills, as reported ratios for Mediterranean annuals very greatly both above and below unity (e.g., Aronson et al., 1992; Garnier, 1992; Padgett and Allen, 1999; Grünzweig and Körner, 2001). To determine biomass, four samples were collected from an area of 0.25 m^2^ each per patch (total 1 m^2^ for B_no_graz_ and 1 m^2^ for B_graz_). B_no_graz_ and B_graz_ were determined at the end of the grazing season and before the onset of litter decay. At each site, B_no_graz_ was determined in ungrazed patches that represented the local vegetation and were available just outside pasture fences. Biomass removal in shrubland included only herbaceous vegetation. Samples were oven dried at 70°C for 72 h, and were then weighed.

### Vegetation Sampling and Trait Measurements

Floristic composition of each plot was determined in four frames of 0.5 m × 0.5 m, which were considered as homogenous and representative samples of the herbaceous vegetation of the respective study plot of 100 m^2^ (Lienin and Kleyer, 2011; Minden et al., 2012). Each frame was subdivided into 25 grid cells of 0.1 m × 0.1 m, and the data from all grid cells of the four frames per plot were pooled over a total area of 1 m^2^. Frequency was defined as the number of grid cells out of the 100 cells where a certain species rooted. In addition, the individuals of each species were counted in each grid cell. In total, 241 mostly annual species were found across all land use types in the Judean Foothills. Floristic composition was mostly (>80% of plots) determined in spring 2007, with a few outstanding plots sampled in 2008.

The presence of a species at two sites at least provided the criterion for its selection for trait measurements. That way, 53 out of the 241 species (see Supplementary Table A1) were used for further analysis of their particular functional traits, whereas for the remainder of the 188 species that occurred at only one site, no trait analysis was performed. Samples for 9 different functional traits (see below) were collected from 10 individuals of the 53 species (for some species, additional individuals had to be collected for seed traits). For measurement of all vegetative and generative functional traits, we selected fully grown individuals at 10 sites of their highest abundance, which presumably represent their optimal habitat. Representative individuals were of average size and at a phenological stage close to seed maturity (but prior to seed shedding). If a species occurred at less than 10 sites, we sampled two or more individuals on each site, until 10 individuals were collected. Trait measurements were performed in 2008, and sampling procedures followed the LEDA Collection and Measuring Standards (Kleyer et al., 2008).

The following groups of traits were assessed in this study: size traits, leaf economic traits, and reproductive traits. Among size traits, we measured canopy height (Can), stem diameter (Sdiam), and leaf biomass (Lbiom) (Pérez-Harguindeguy et al., 2013). Sdiam and Can were determined on vegetative structures in the field (regarding grasses, stem diameter included culm and leaf sheaths). To determine Lbiom, all leaves of one individual plant were dried to constant mass (60°C) and were then weighed (“leaves” of grasses included leaf blades only). Leaf economic traits included specific leaf area (SLA; leaf area per leaf mass), leaf dry matter content (LDMC), leaf nitrogen content (LNC), and nitrogen-to-phosphorus ratio (LNP). Finally, reproductive traits included SM (the mass of a single seed) and SN (the number of seeds per plant).

Samples for leaf, stem, and seed traits were transported to the lab on ice. Leaf trait analysis based on 20 mature and fully expanded leaves (two leaves per sampled individual) located in the middle part of the stem. Leaf area and leaf fresh weight were measured on water-saturated leaves. Leaf area analysis was performed on leaf scans with a resolution of 600 dpi by using the freeware ImageJ^1^.

Leaf dry mass (60°C to constant mass) was taken to determine SLA and LDMC. Dry leaves were then pooled and ground (Pulverisette 7 Planetary Micro Mill, Fritsch, Idar-Oberstein, Germany) to measure leaf carbon content and LNC on duplicated samples in an elemental analyser (Model EA 1108, Carlo Erba Instruments, Milan, Italy). For leaf phosphorus content, ground leaf material (0.15 g) was treated by 65% nitric acid overnight and then incubated in 90°C for 4 h. Solutions were analyzed for phosphorus on an ICP-OEP (Spectro Arcos, Kleve, Germany). SM is the mass of one mature seed after removing dispersal structures as carefully as possible. To determine SM, 10 seeds were weighed for each of the 10 individuals. Total SN was reported per individual. For species with very small seeds and extremely high SN, a total number of approximately 400 seeds was counted from 5 to 10 fruits for each individual. Then SN was calculated according to the total number of fruits per individual. The final value of each trait allocated to a species was averaged over the 10 sampled individuals.

### Soil Analysis

Soil profiles were often shallow, thus soil analyses were based on five samples randomly taken by a soil driller (0.05-m diameter) to 0.2 m depth at each plot. Soil samples were air dried, bulked and sieved to 2 mm, with visible plant remains being removed. For soil texture analysis, 40 g soil per sample were dispersed by adding a sodium hexametaphosphate solution and mixing the sample for 4 h. Texture was determined with the hydrometer technique (Klute, 1986). Concentration of soil organic carbon (SOC), total soil nitrogen (TN), and the soil C/N ratio (C/N) were determined with an elemental analyser (FlashEA 1112; Thermo, Waltham MA, United States) after treating samples with 1 N HCl to remove carbonates (Midwood and Boutton, 1998). Soil phosphorus availability (P) was analyzed according to Olsen and Sommer (1982). Soil water holding capacity (WHC) was estimated from soil texture at field capacity using a hydraulic properties calculator^2^. Soil mass was expressed on 105°C oven-dry base.

### Data Analysis

To reveal the covariation between plant functional traits and environmental variables, we performed an RLQ analysis (Dolédec et al., 1996), which links functional traits and environmental variables by a species abundance table in a three-way ordination. In this method, the statistical units are the species with their individual abundances in each study plot (frequency over the 100 grid cells), as opposed to approaches using community weighted mean traits where the community is the statistical unit and trait values of all species of a community are averaged. Species-level methods such as RLQ can better address functionally diverse species communities, which represent different survival strategies or different ways of resource exploitations in a community (Kleyer et al., 2012). In community ecology, RLQ techniques were successfully used to relate traits to the main sources of environmental variability for both plant (Thuiller et al., 2006; Minden et al., 2012) and animal assemblages (Ribera et al., 2001; Lindo et al., 2012). Following the RLQ analysis, we performed a fourth corner analysis (Legendre et al., 1997), which tests for individual relations between traits and environmental variables. Generally, RLQ and fourth corner analyses are based on similar mathematical principles, but they have different objectives and outputs: whereas the RLQ analysis produces a simultaneous ordination of the three tables, the fourth corner analysis combines the three tables into a matrix to evaluate significant trait-environment associations.

Collinearity of functional traits (Can, Sdiam, Lbiom, SLA, LDMC, LNC, LNP, SM, and SN) and environmental factors (Dist, SOC, CN, P, and WHC) was <0.8. RLQ achieves the link between traits and environment by performing a double inertia analysis of two arrays, the R-matrix (a site-environment file) and the Q-matrix (a species-trait file, using the 53 species used for trait analysis), with a link expressed by a contingency table of species abundance, the L-matrix (a site-species file) (Dolédec et al., 1996). Prior to the RLQ analysis, a separate ordination was performed on each of the three tables. The L-matrix, containing information about species abundance was analyzed by correspondence analysis (CA). The R-matrix and the Q-matrix, containing information about environmental variables (Dist, P, SOC, C/N, and WHC) and functional traits (Can, Lbiom, Sdiam, SLA, LDMC, LNC, LNP, SM, and SN), respectively, were each analyzed by a principal component analysis (PCA).

The RLQ then combined the R- and Q-matrices with row weights (field sites) and column weights (species abundance) of the L-matrix into one analysis by extracting axes that maximize the covariance between traits and environmental factors through the link of the species abundance by the L-matrix (Dolédec et al., 1996). The structure of the individual tables can only be partially optimized owing to the constraints imposed by the joint analysis. The RLQ axes compromise between maximizing the correlation and explaining the variation of each matrix, also referred to as co-inertia. The Eigen values of the joint analysis do not represent the variance of the scores, as in the separate analysis, but their squared covariances (Dolédec and Chessel, 1994) between functional traits and environmental factors. The fourth corner analysis of the ordination was applied to test the statistical significance of relationships between functional traits and environmental variables (Dray and Legendre, 2008).

To test for significant trait-environment relations, we used the fourth corner approach of Dray et al. (2014). To test the H0 hypothesis that species traits (Q-matrix) are unrelated to the characteristics of the sites (R-matrix), we used the methods of permuting the L-matrix (sites vs. species) according to Dray and Legendre (2008). The following permutations were applicable: Model 2 is equivalent to permuting the rows of the R-matrix, which removes the link between L- and R-matrix, but keeps L-linked to Q-matrix (H1: R ↔ L). Model 4 is equivalent to permuting the columns of the L-matrix or rows of the Q-matrix. This removes the link between L- and Q-matrix, but keeps L-linked to R-matrix (H1: L ↔ Q). The combination of the permutation Models 2 and 4 (Model 6) eliminates the so far undissolved critical issue of different units between species and samples that was addressed in Dolédec et al. (1996) and Dray and Legendre (2008). Model 6 bases on two sequential tests and performs the second test only, if the null hypothesis is rejected by the first test (Ter Braak et al., 2012; Dray et al., 2014). By using the sequential approach, the association between a trait and an environmental variable is considered significant, if the highest of the two probability values (obtained from Models 2 and 4) is lower than 0.05. The performance of the fourth corner analysis further considered the issue that a higher number of tests for significance also increases the chance of potential significant relations. In the current study, 9 functional traits and 5 environmental variables added up to 45 possible significant trait-environment relations. Therefore, the high number of 49,999 permutations was used, and probability values were adjusted by the false discovery rate (FDR) of Benjamini and Hochberg (1995). The species scores of the first two RLQ axes were used to define plant functional groups using Ward’s hierarchical clustering (Everitt et al., 2001) that tends to form compact, spherical clusters of similar size (Legendre and Legendre, 1998). The optimal number of cluster was determined by the Calinski-Harabasz index (Gordon, 1999).

Trait and environmental variables were log-transformed prior to the analyses. We refrained from incorporating phylogenetic relatedness in our analysis mainly because we missed a phylogenetic tree of species found in our study area. Combining trait and phylogenetic analyses could potentially show the extent of phylogenetically independent trait convergence versus phylogenetic trait conservatism (Pavoine et al., 2011). All separate ordination analyses, cluster analysis, RLQ statistics, and subsequent significance tests were performed using the ade4 package (Dray and Dufour, 2007) in the open source software R (R Development Core Team, 2008).

## Results

### Species Composition along the Disturbance Gradient

**FIGURE 1:**
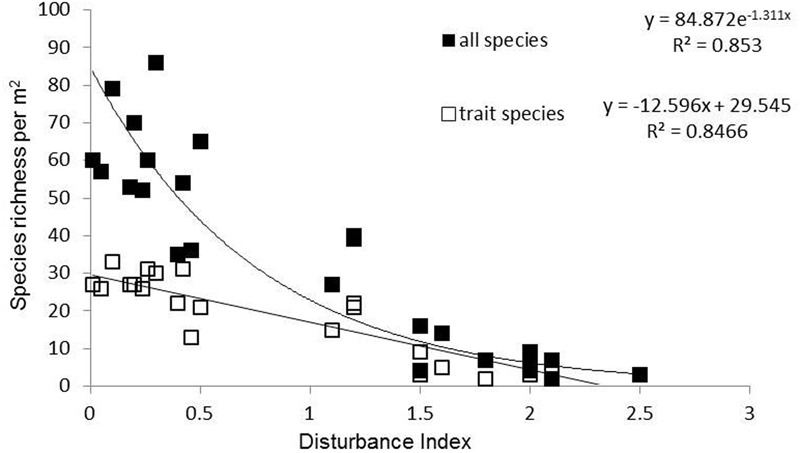
Species richness (total number of species) and number of trait species per m^2^ along the disturbance gradient in the Judean Foothills.

The land use gradient in the Judean Foothills spanned very low to very high disturbance intensities (Dist = 0.01–2.50) (**Figure 1** and **Table 1**). Among the total of 241 mostly annual species that were identified along the entire gradient, 30 of them were grasses, 54 were legumes, and 157 were other forbs. Species richness declined along the disturbance gradient, from 65 species per m^2^ on average in ungrazed natural shrubland (Dist = 0.01–0.1) to 5 species per m^2^ in cropland cultivated with cereal (Dist = 1.5–2.5; **Figure 1** and **Table 1**). Accordingly, the number of species selected for functional trait analysis decreased from 29 species in ungrazed shrubland to 3 species on cropland cultivated with legumes. The relative abundance of species selected for functional trait analysis averaged 56% based on species richness and 69% based on the number of individuals, and did not vary considerably among land use types (data not shown).

Sites and land use types spread not only along a disturbance gradient, but also along a range of other environmental factors (**Table 1**). Highly to moderately disturbed agricultural fields and olive groves had low SOC stocks, but relatively high soil C/N ratios as a result of greatly reduced TN stocks. Grassland and cereal cropland showed relative high soil P. WHC varied little among land use types.

### Relationships among Plant Functional Traits and Environmental Factors

**FIGURE 2:**
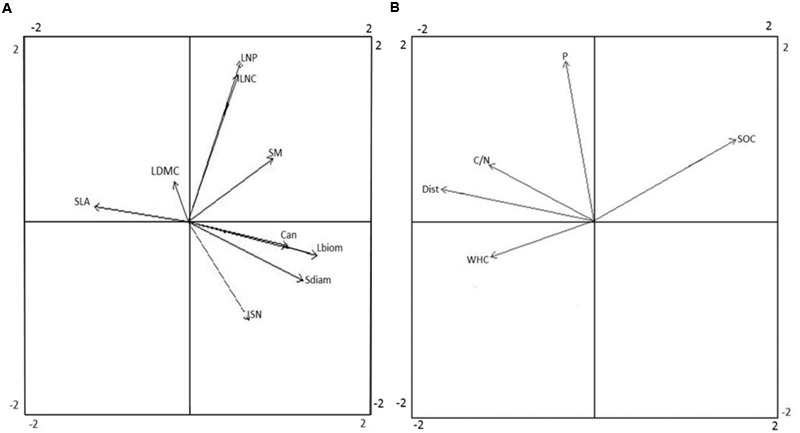
Principal component analysis (PCA) of plant functional traits **(A)** and environmental factors **(B)**. Arrow lengths indicate the correlation between trait or environmental variables and ordination axes. Eigenvalues for axis 1 and 2 were 1.78 and 1.30 for **(A)** and 1.48 and 1.09 for **(B)**, respectively. The ordination explained 53.8% of the variation in the data set by the first two axes (35.1 and 18.7%, respectively) in **(A)**, and 68.0% of the variation in the data set (44.0 and 24.0%) in **(B)**. Acronyms for functional traits **(A)**: Can, canopy height; Lbiom, leaf biomass; LDMC, leaf dry matter content; LNC, leaf nitrogen content; LNP, leaf nitrogen-to-phosphorus ratio; Sdiam, stem diameter; SLA, specific leaf area; SM, seed mass; SN, seed number. Acronyms for environmental factors **(B)**: C/N, soil carbon-to-nitrogen ratio; Dist, disturbance; P, soil phosphorus availability; SOC, soil organic carbon stock; WHC, soil water holding capacity.

Separate PCAs were conducted to show the respective correlation structure of plant functional traits and environmental factors across study plots. The traits representing plant size (Can, Sdiam, and Lbiom) co-varied largely along the first PCA axis of plant traits, were negatively related to SLA, and were positively related to SN and to some extent also to SM (**Figure 2A**). Among the leaf economic traits, SLA was not clearly related to either LNC or LNP, and all of these traits co-varied slightly with LDMC. Among the reproductive traits, SN was not clearly related to SM. The leaf economic traits exhibited low variation among plots, with the coefficient of variance (CV) ranging from 0.12 to 0.20, compared to the considerably higher variation in traits related to plant size (CV 0.36–0.85) and reproductive traits (CV 0.57–0.69; Supplementary Figure A2). In the PCA of environmental factors, soil C/N ratio, Dist, and WHC co-varied along the first axis, and were negatively related to SOC stock (**Figure 2B**). Soil P was the main factor related to the second axis of the PCA.

### Trait-Environment Relationships

**FIGURE 3:**
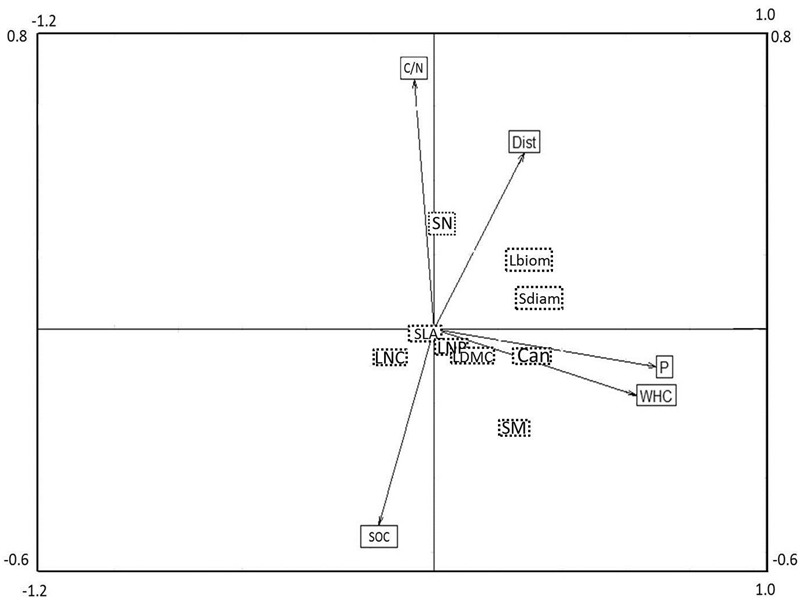
Co-variation of plant functional traits and environmental factors in a joint RLQ analysis. The position of plant traits can be found in the center of the respective boxes (dashed line, no arrows), whereas the position of environmental variables in the ordination is indicated by the end point of the respective arrows. Eigen values for axis 1 and 2 were 0.39 and 0.21, covariance of the axes were 0.63 and 0.45, respectively. For a comparison of the results of the related fourth corner analysis, see **Table 2**. For acronyms of plant functional traits and environmental variables, see **Figure 2**.

The combined analysis of species, traits and environmental factors (RLQ analysis) revealed that traits indicating plant size (Can, Sdiam, and Lbiom) and reproduction (SM) could be linked to soil P and WHC, and possibly to Dist (**Figure 3**). Leaf economic traits did not show any clear relationship to environmental factors. The fourth corner analysis tested the significance of trait and environmental factors with the respective RLQ axis. The first axis was significantly positively associated with all traits indicating plant size and the resources soil P and WHC; SM was marginally positively related to axis 1 (**Table 2**). Axis 2 was significantly positively related to the reproductive trait SN and to soil C/N ratio, negatively to SOC, and marginally positively to Dist. The leaf economic traits (SLA, LDMC, LNC, and LNP) did not significantly correlate with any RLQ axis. The first two axes of the RLQ analysis explained 94.8% of the total co-structure of traits and environmental variables. The proportion of variance was 0.62 (axis 1) and 0.79 (axis 2) for traits and 0.96 (axis 1) and 0.6 (axis 2) for environmental factors compared to those obtained by the respective separate analyses.

**Table 2:** Probability values of the fourth corner analysis to test for significant relations between RLQ axes and functional traits or environmental variables.

Variable	Axis 1	Axis 2
**Functional traits**		
Can	**0.014**	0.724
Lbiom	**0.015**	0.256
Sdiam	**0.008**	0.724
SLA	0.906	0.906
LDMC	0.456	0.724
LNC	0.417	0.724
LNP	0.723	0.838
SM	0.072	0.106
SN	0.906	**0.042**
**Environmental factors**		
Dist	0.401	0.060
SOC	0.632	***0.034***
C/N	0.790	**0.006**
P	**0.004**	0.750
WHC	**0.008**	0.632

*Bold values in normal style indicate a significant positive correlation at *p* ≤ 0.05, bold values in italics indicate a significant negative correlation at *p* ≤ 0.05, underlined values indicate a marginally positive correlation at 0.05 < *p* < 0.1. For acronyms of plant functional traits and environmental variables, see **Figure 2**.*

### Cluster Analysis and Plant Functional Groups

**FIGURE 4:**
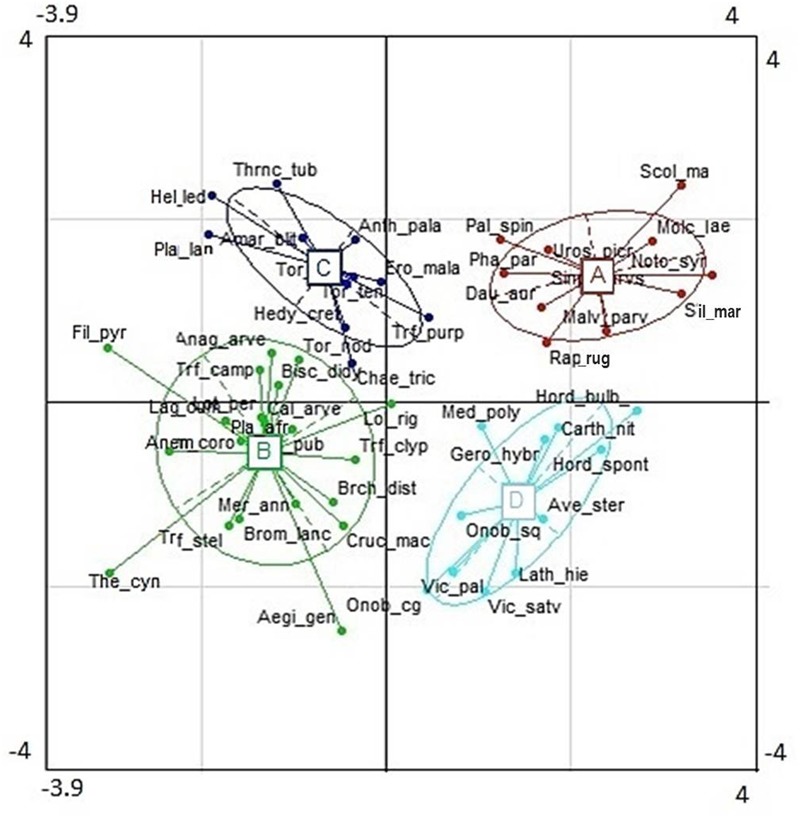
Functional groups obtained from cluster analysis along the first two RLQ axes. For species abbreviation, see Supplementary Table A1.

**FIGURE 5:**
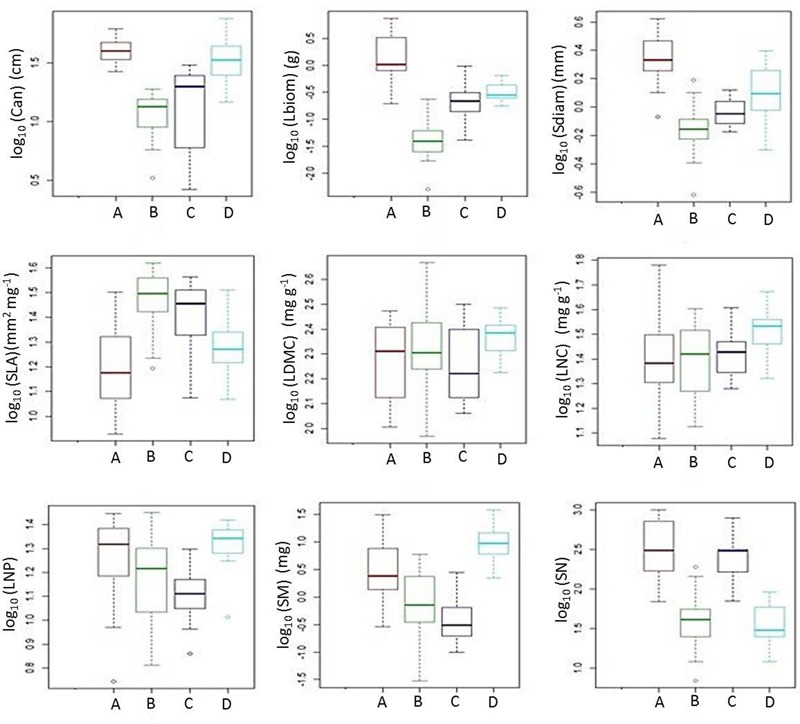
Box plots illustrating quantitative plant traits of functional groups A-D (see **Figure 4**). The figure shows the median among species traits (bold line), 25–75% quartiles (boxes), and <1.5 times the interquartile range (whiskers). Open circles show extreme outliers exceeding 1.5 times the interquartile range. For acronyms of plant functional traits, see **Figure 2**.

The cluster analysis defined four distinct groups consisting of 10–19 out of the 53 species for which plant functional traits were determined (**Figure 4**). These clusters (functional groups) were calculated on the scores of the first and second RLQ axis. Short grasses, legumes and forbs with low Lbiom and Sdiam formed a rich and diverse group (group B) that occurred on the most natural sites where both Dist and soil P tended to be low and SOC tended to be high (**Figures 3, 5**). Plants belonging to functional group B had high SLA and low SN. SM peaked in group D, with mostly legumes, but also grasses and forbs growing at high soil P, high WHC, and low Dist. These tall plants had a low SN that was similar to mean SN of group B, which occurred also under low Dist. Under high levels of Dist and resource availability (soil P and WHC) (group A), plants grew taller and bigger, and had more and heavier seeds, whereas SLA was lower compared to the community at natural field sites (group B). Medium sized plants in group C grew under relatively high Dist, but low resources, and were characterized by high SLA, high SM, and low SN.

## Discussion

The dry conditions of the Judean Foothills, with an extended rainless period of 5 months or more promote the predominance of annuals among the herbaceous life forms of this species-rich Mediterranean landscape. While annual life form is an adaption to extreme drought during summer months, the species composition of annuals changed along the broad land use gradient. These changes were associated with shifts in plant functional traits within a matrix of site-specific environmental factors. Functional traits indicating plant size co-varied positively with the soil resources P availability and WHC, while the reproductive trait SN was positively related to disturbance and soil C/N ratio and negatively to SOC stocks. However, the major leaf economic traits (SLA, LDMC, LNC, and leaf N/P ratio) were not significantly related to any of the assessed variables of disturbance intensity or resource availability, and the variation among species in those traits was rather small. With respect to potential drivers of community composition in the Mediterranean annual vegetation under varying land use intensity, light interception, competition, and dispersal might thus be more relevant than nutrient absorption and turnover, and litter decomposition.

### Disturbance-Resource Relationships

Along the whole land use gradient, which comprised ungrazed and grazed shrubland, grazed grassland, and more intensively managed olive groves and cropland, disturbance intensity was positively associated with soil WHC and soil C/N, but negatively with SOC and remained unrelated to soil P. This pattern contrasts with results from temperate regions and wetlands where resource availability often increases with increasing land use intensity (McIntyre, 2008; Culman et al., 2010; Lienin and Kleyer, 2011). One possible explanation for this pattern arises from the fact that grazers add readily available nutrients to soils (Hobbs, 1996), especially under conditions of supplemental feeding. Grazing increased rates of nutrients mineralization, cycling and root-uptake in temperate grassland of Argentina (Chaneton et al., 1996). This can explain the relatively high P availability and low soil C/N at grazed (i.e., moderately disturbed) grassland sites.

Legume crops use large amounts of P (Grünzweig and Körner, 2003), thus potentially explaining why P availability was low under the highly disturbed and N self-sufficient legume crops. Furthermore, soil moisture and precipitation play a fundamental role for understanding shifts in nutrient cycling as affected by grazing. Forbs with a high leaf N/P ratio increased under grazing in wet sites, whereas in intermediated and dry sites, species supported by grazing had a lower leaf N/P ratio than those diminished by grazing (Semmartin et al., 2004), thus indicating high soil P as in grasslands of the current study.

### Trait-Environment Relations

High soil P and soil WHC promoted taller plants and associated support structure, such as stem diameter. These patterns indicate trait convergence toward stronger competitive ability on favorable sites (Grime, 2001; Lehsten and Kleyer, 2007; Poorter et al., 2012). Traits related to plant size suggest light interception and competitive ability as drivers of community composition (Lavorel et al., 2007). The positive relation between size-related traits, and soil P and soil WHC indicates that those drivers were relevant, even when field sites were more disturbed. This can be seen in the context of Coley’s resource availability hypothesis and Lind’s growth defense hypothesis, which convey a faster growth, a larger leaf turnover and a generally higher tolerance to herbivory on more fertile sites (Coley et al., 1985; Lind et al., 2013). Although functional group A was characterized by large plant size and comprised the species more abundant on sites exposed to cattle grazing and croplands, the overall pattern did not relate traits associated with plant size to disturbance intensity, thus refuting our first hypothesis.

None of the leaf economic traits varied with disturbance intensity or resource availability, which rejects our second hypothesis. In other biomes, particularly in temperate regions, leaf economic traits were found to respond strongly to land use gradients. In a study of similar scope and methodology to the current one, SLA and LNC significantly increased with land use intensity and soil fertility in temperate communities (Lienin and Kleyer, 2011). The lack of relationships between leaf economic traits and environmental factors in our Mediterranean communities needs to be assessed against the fact that the annual life form was dominant in this herbaceous vegetation, whereas it is predominantly perennial in many other regions. Many herbaceous plant species growing on shallow, infertile soils in shrubland were small ephemeral annuals with a short, drought-limited life span. This growth form probably represents a temporal grazing and drought escape strategy (Hadar et al., 1999; Milchunas and Noy-Meir, 2002). Along a climate gradient ranging from Mediterranean rangelands to subalpine grasslands in north-eastern Spain, grazing favored ephemeral species particularly under dry conditions (De Bello et al., 2005). Short life span often requires high SLA to rapidly reach the reproductive stage and, therefore, tends to be found mainly at the acquisitive part of the leaf economics spectrum (Reich et al., 1997; Wright et al., 2004). Moreover, their reproductive effort is usually higher than that of perennial species (Obeso, 2002). Among semi-natural and agricultural grasslands in Scotland, grazing intensification induced a shift from perennial to annual life forms that favored a suite of attributes associated with short life spans, fast regeneration and growth, seasonal regeneration by seed, and a ruderal strategy (Pakeman, 2004). The dry climatic conditions in our study likely filtered the species to be located in proximity on the leaf economics spectrum (resulting in low variability of leaf economic trait values among species), with little possibility of the regional species pool to respond to disturbance and resources. This low variation in leaf economic traits likely precluded co-variation with environmental factors in these Mediterranean ecosystems. By analogy, a weak response of canopy height to grazing was found in subalpine grasslands because variation in this trait was small as low canopy is an adaptation to the climate conditions of a high altitude ecosystem (De Bello et al., 2005). This confirms the fundamental role of both climate conditions and the predominant growth forms of the regional species pool for understanding trait responses to environmental factors.

The above results hold information about potential drivers of community composition in this annual Mediterranean vegetation. Resource acquisition mechanisms, as reflected by leaf economic traits, seem not to represent relevant drivers of community composition in this study. This becomes also obvious from the low variation in litter decomposition during summer among the herbaceous sites of this study (Dirks et al., 2010). Instead, the high variability of trait values related to plant size reveals competition as an important driver of community composition that becomes even more relevant under high soil P availability.

Increasing land use intensity and resource availability promoted reproductive traits, SN under high disturbance (confirming our third hypothesis), SM under high soil P and soil WHC. Higher SN increases the number of multiple colonization opportunities (Leishman, 2001), which is advantageous under unstable, disturbed conditions. High resource availability appeared to promote species with high SM, possibly because high SM facilitates establishment under conditions of heavy shading among plants. In various ecosystems across Europe, SM increased with vegetation height, but was independent of soil fertility (Pakeman et al., 2008). The higher SM on P rich sites can be additionally related to larger plant size on more fertile sites.

### Relationships among Leaf Economic Traits

Regarding trait-trait relationships, SLA was not related to LNC, which was unexpected because both traits are usually strongly positively correlated on landscape and global scales (Wright et al., 2004). The increasing need for defense against grazing with increasing disturbance in the lower part of the land use gradient possibly prevented a positive relationship of SLA with LNC and enabled a slightly positive relationship between LNC and LDMC. To defend their leaves against grazing, plants allocate an increasing proportion of leaf N to non-photosynthetic compounds (Mustard et al., 2003). Thus, some high-LNC species might invest proportionally less into their photosynthetic apparatus and into a large leaf area, but more into N-containing secondary metabolites. Moreover, defense traits, such as spines on leaves often imply small leaf size and high LDMC (Wardle et al., 1998; Díaz and Cabido, 2001), thus leading to low palatability and reduced SLA at grazed sites of dry climates. Low SLA in combination with high LNC was further detected among plants negatively affected by grazing intensity in semiarid grasslands (Li et al., 2016). Under more moist conditions, grazing-tolerance traits, e.g., shoot resprouting ability, maintain a positive relation between SLA and LNC (Adler et al., 2004; Cingolani et al., 2007). However, trait correlations among succulents and salt marsh plants also deviated from predictions by the leaf economics concept (Vendramini et al., 2002; Minden et al., 2012). The current study indicates that adaptation to a long grazing history by investments in N-rich deterrents may add another exception to the general acceptance that SLA and LNC are strongly correlated.

### Relationships among Reproductive Traits

No clear trade-off between SN and SM was obvious across our land use gradient, although empirical evidence in temperate regions would support such a trade-off in species of similar size (Jakobsson and Eriksson, 2000; Kühner and Kleyer, 2008). The relationship between the two reproductive traits can be exemplified by the four functional groups in our study. Functional groups C and D were of similar size and showed the expected low seed mass and high SN (group C) or vice versa (group D). However, functional group A was characterized by relatively high SM and high SN, as well as high values of size traits. High SN and SM in this group were potentially enabled by the large size of its species (Lehsten and Kleyer, 2007). Accordingly, functional group B, which was composed of small plants, had low SM and SN. Therefore, the lack of a SM-SN trade-off in this study could be caused by the relatively large difference in size of the herbaceous plants and, thus, in their capacity to produce seeds (Moles and Westoby, 2006). In addition, SN was related to disturbance, and SM to soil P and soil WHC, with the two resources not being closely related to disturbance. Thus, the low relation between resources and disturbance might further explain the lack of a trade-off between SM and SN.

## Conclusion

Functional traits indicating plant size and reproduction but not leaf economic traits were related to land use and environmental factors in this semiarid annual vegetation. These results considerably differ from more mesic ecosystems, where leaf economics traits were related to disturbance and soil resources. Consequently, life history and climate conditions play a fundamental role for understanding trait responses to land use intensity. Testing the variability of multiple traits can be advantageous before testing single trait responses to environmental factors in distinct study regions.

As many temperate regions experience increasingly warm and dry periods, a filtering of life forms, which in turn may reduce variability in leaf economic traits and make them less relevant might occur in herbaceous communities. Prospectively, community composition of the herbaceous vegetation might be increasingly driven by light interception, competitive availability and recruitment. Nutrient deposition, as a result of land use intensification, might enhance the presented shifts in functional traits and drivers of community composition. Future research should focus on interrelations between drought, disturbance and resource availability to better understand factors that control herbaceous community composition under global change.

## Author Contributions

JG and MK conceived and designed the study; ID and RD performed the research; ID and PL analyzed the data; ID, MK, and JG wrote the paper.

## Conflict of Interest Statement

The authors declare that the research was conducted in the absence of any commercial or financial relationships that could be construed as a potential conflict of interest.

## Footnotes

1 https://imagej.nih.gov/ij/

2 www.pedosphere.com/resources/texture/triangle_us.cfm
