# Supplementary material for: Size and Reproductive Traits Rather than Leaf Economic Traits Explain Plant-Community Composition in Species-Rich Annual Vegetation along a Gradient of Land Use Intensity
Source: Front Plant Sci. 2017 May 29;8:891. doi: 10.3389/fpls.2017.00891 (PMC5447063; doi:10.3389/fpls.2017.00891)
Supplement: Supplementary file 1 [file Data_Sheet_1.DOCX]

**A1**

**
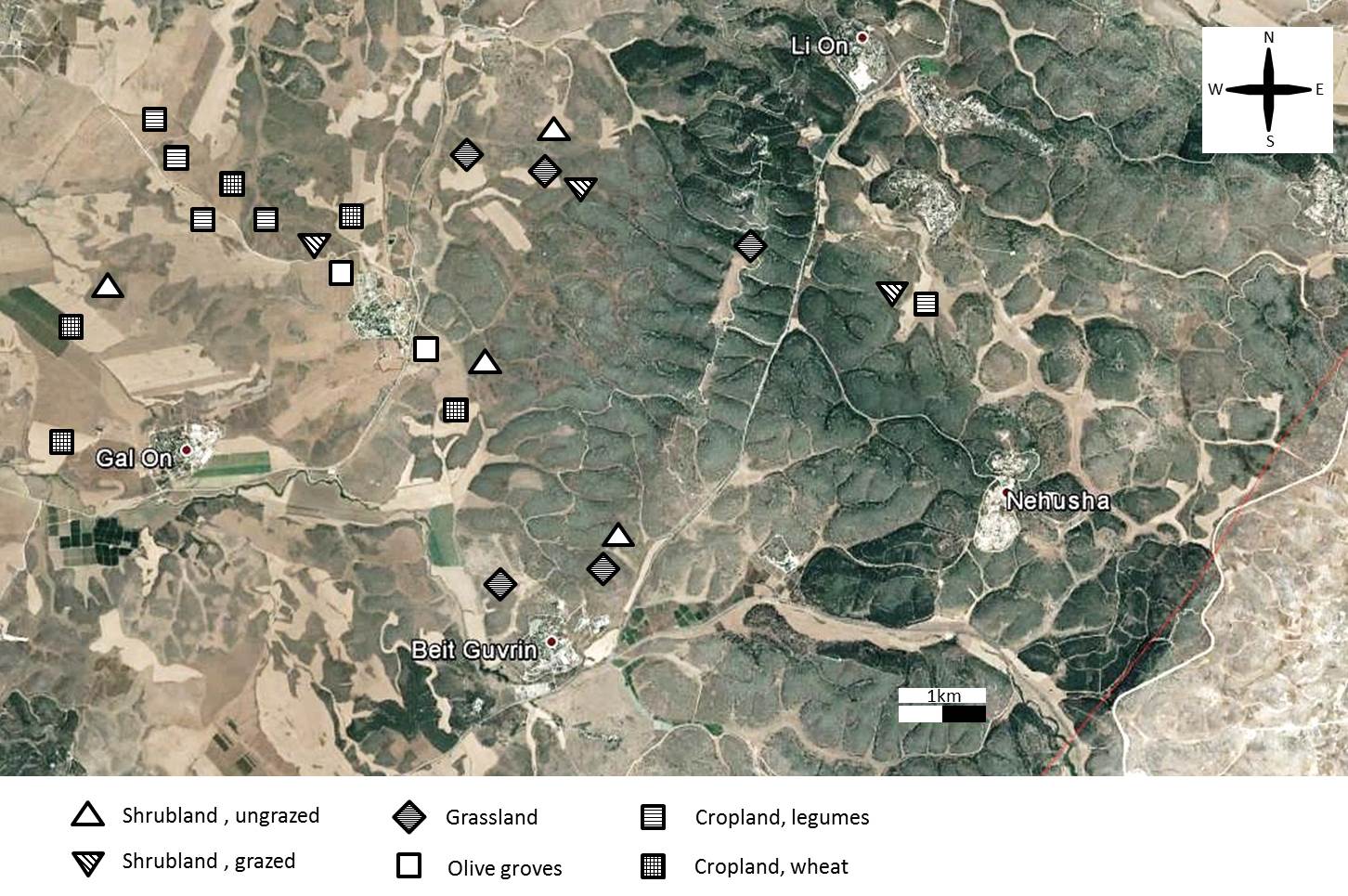
**

Fig. A1. Map representing field sites in the Judean Foothills, central Israel**.** The map was generated by using Google Earth (2016).

**A2**


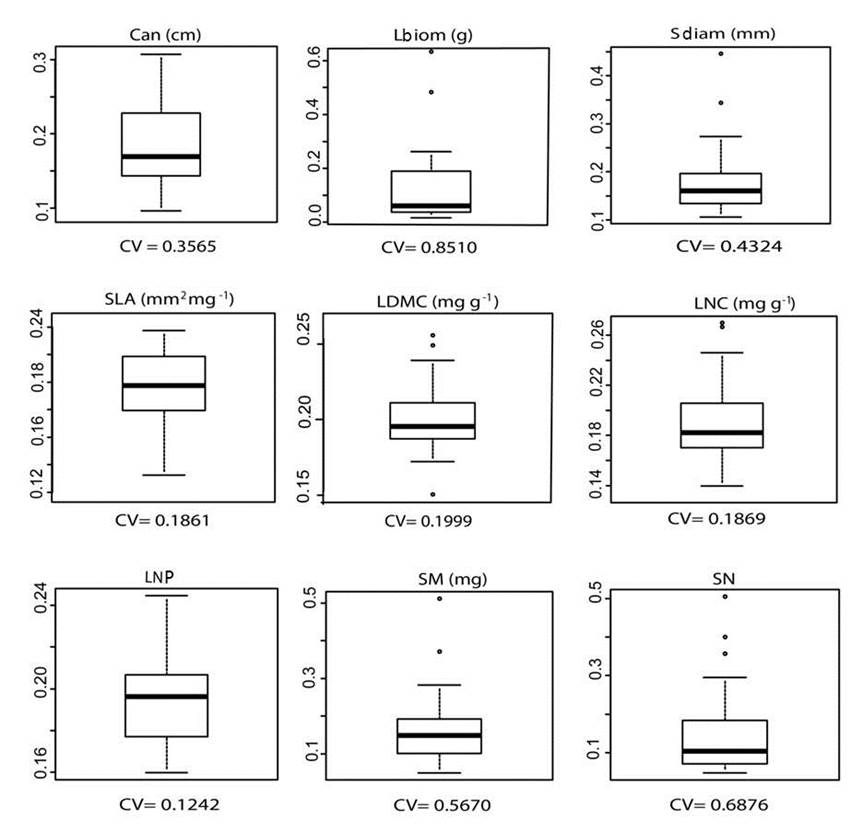


Fig. A2. Box plots illustrating quantitative plant traits of standardized community weighted means of all study plots. The figure shows the median among species traits (bold line), 25% to 75% quartiles (boxes), and <1.5 times the interquartile range (whiskers). Open circles show extreme outliers exceeding 1.5 times the interquartile range. For acronyms of plant functional traits, see Fig 2. The coefficient of variation (CV) is shown for each functional trait.
