# Supplementary material for: Size and Reproductive Traits Rather than Leaf Economic Traits Explain Plant-Community Composition in Species-Rich Annual Vegetation along a Gradient of Land Use Intensity
Source: Front Plant Sci. 2017 May 29;8:891. doi: 10.3389/fpls.2017.00891 (PMC5447063; doi:10.3389/fpls.2017.00891)
Supplement: Supplementary file 2 [file Data_Sheet_2.docx]

**Supplementary table**

Table A1. ‘Trait’ species, with life form and affiliation to functional groups (FG) in the cluster analysis (Fig. 4).

| Species | Abbreviation | Family | Life form | FG |
| --- | --- | --- | --- | --- |
| *Aegilops geniculata* | Aegi_gen | Poaceae | annual | B |
| *Amaranthus blitoides* | Amar_blit | Amaranthaceae | annual | C |
| *Anagallis arvensis* | Anag_arve | Primulaceae | annual | B |
| *Anemone coronaria* | Anem_coro | Ranunculaceae | geophyte | B |
| *Anthemis palaestina* | Anth_pala | Asteraceae | annual | C |
| *Avena sterilis* | Ave_ster | Poaceae | annual | D |
| *Biscutella didyma* | Bisc_didy | Brassicaceae | annual | B |
| *Brachypodium distachyon* | Brch_dist | Poaceae | annual | B |
| *Bromus lanceolatus* | Brom_lanc | Poaceae | annual | B |
| *Calendula arvensis* | Cal_arve | Asteraceae | annual | B |
| *Chaetosciadium trichospermum* | Chae_tric | Apiaceae | annual | C |
| *Crucianella macrostachia* | Cruc_mac | Rubiaceaec | annual | B |
| *Carthamus nitidus* | Carth_nit | Asteraceae | annual | D |
| *Daucus aureus* | Dau_aur | Apiaceae | annual | A |
| *Erodium malacoides* | Ero_mala | Geraniaceae | annual | C |
| *Filago pyramidata* | Fil_pyr | Asteraceae | annual | B |
| *Geropogon hybridum* | Gero_hybr | Asteraceae | annual | D |
| *Hedypnois cretica* | Hedy_cret | Asteraceae | annual | C |
| *Helianthemum ledifolium* | Hel_led | Cistaceae | annual | C |
| *Hordeum bulbosum* | Hord_bulb | Poaceae | hemicrypt. | D |
| *Hordeum spontaneum* | Hord_spon | Poaceae | annual | D |
| *Lagoecia cuminoides* | Lag_cum | Rosaceaea | annual | B |
| *Linum pubescens* | Lin_pub | Linaceae | annual | B |
| *Lathyrus hierosolomitata* | Lath_hie | Fabacea | annual | A |
| *Lolium rigidum* | Lol_rig | Poaceae | annual | B |
| *Lotus peregrinus* | Lot_per | Fabaceae | annual | B |
| *Malva parviflora* | Malv_parv | Malvaceae | annual | A |
| *Medicago polymorpha* | Med_ poly | Fabaceae | annual | D |
| *Mercurialis annua* | Mer_ann | Euporbiaceae | annual | B |
| *Moluccella laevis* | Molc_lae | Lamiaceae | annual | A |
| *Notobasis syriaca* | Noto_ syr | Asteraceae | annual | A |
| *Onobrychis crista-galli* | Onob_cg | Fabaceae | annual | D |
| *Onobrychis squarrosa* | Onob_sq | Fabaceae | annual | D |
| *Pallenis spinosa* | Pal_spin | Asteraceae | annual | A |
| *Phalaris paradoxa* | Pha_par | Poaceae | annual | A |
| *Plantago afra* | Pla_afr | Plantaginaceae | annual | B |
| *Plantago lanceolata* | Pla_lan | Plantaginaceae | annual | A |
| *Rapistrum rugosum* | Rap_rug | Asteraceae | annual | A |
| *Scolymus maculatus* | Scol_ma | Asteraceae | annual | A |
| *Silybum marianum* | Sil_mar | Asteraceae | annual | A |
| *Sinapis arvensis* | Sin_arvs | Brassicaceae | annual | A |
| *Theligononium cynocrambe* | The_cyn | Theligonaceae | annual | B |
| *Thrincia tuberosa* | Thrnc_tub | Asteraceae | annual | C |
| *Torilis arvensis* | Tor_arv | Apiaceae | annual | C |
| *Torilis nodosa* | Tor_nod | Apiaceae | annual | C |
| *Torilis tenella* | Tor_ten | Apiaceae | annual | C |
| *Trifolium campestre* | Trf_camp | Fabaceae | annual | B |
| *Trifolium clypeatum* | Trf_clyp | Fabaceae | annual | B |
| *Trifolium purpureum* | Trf_purp | Fabaceae | annual | C |
| *Trifolium stellatum* | Trf_stel | Fabaceae | annual | B |
| *Urospermum picroides* | Uros_picr | Asteraceae | annual | A |
| *Vicia palaestina* | Vic_pal | Fabaceae | annual | D |
| *Vicia sativa* | Vic_satv | Fabaceae | annual | D |
